# Analysis of the completeness of self-harm and suicide records in Pernambuco, Brazil, 2014–2016

**DOI:** 10.1186/s12889-022-13455-8

**Published:** 2022-06-09

**Authors:** Jéssica Ramalho da Fonsêca, Conceição Maria de Oliveira, Cláudia Cristina Lima de Castro, Heitor Victor Veiga da Costa, Pauliana Valéria Machado Galvão, Albanita Gomes da Costa Ceballos, Cristine Vieira do Bonfim

**Affiliations:** 1 Postgraduate Program in Collective Health, Federal University of Pernambuco, Recife, PE Brazil; 2 Maurício de Nassau University Center, Health Secretary of Recife, Recife, PE Brazil; 3 Health Secretary of Recife, Recife, PE Brazil; 4 Postgraduate Program of the Informatics Center, Federal University of Pernambuco, Recife, PE Brazil; 5 School of Medicine, University of Pernambuco, Serra Talhada, PE Brazil; 6 Directorate of Social Research, Joaquim Nabuco Foundation, Rua Dois Irmãos, 92 - Ed. Anexo Anísio Teixeira – Apipucos, Recife, PE CEP 52071-440 Brazil

**Keywords:** Suicide, Attempted suicide, Record completeness, Record linkage, Health information systems

## Abstract

**Introduction:**

Suicides and suicide attempts are major public health problems, and coping strategies are hampered by insufficient or inadequate notifications. Data accuracy influences the formulation of public and mental health policies and suicide prevention strategies. The objective of this study was to analyze the completeness of self-harm and suicide records in the state of Pernambuco, Brazil, 2014–2016.

**Methods:**

This is an evaluative study with a descriptive design. The data were collected from suicide attempt records from the Notifiable Diseases Information System and suicide records from the Mortality Information System. Probabilistic linkage was used to relate these databases, and the degree of completeness of the variables was calculated. Completeness was classified into the following categories: good (≥ 75.1%), regular (50.1%–75.0%), low (25.1%–50.0%), and very low (≤ 25.0%).

**Results:**

In the analyzed period, 1,404 notifications of self-harm were studied, with an overall mean completeness of 86.2%. In addition, 1,050 suicide records were analyzed, with an overall mean completeness of 95.8%. Most variables referring to suicide attempts had good completeness, with the exception of the variables “occupation” and “education.” The completeness of all suicide-related variables was rated as good. After linkage, a significant improvement was observed in the degree of completeness of the variable “occupation”.

**Conclusion:**

The results of this study showed that the completeness of self-harm and suicide variables improved from the first to the last year. The integration of data from different information systems provides an opportunity to improve suicide prevention programs and the quality of available information. Continuous efforts to increase the completeness and reliability of suicide surveillance systems are fundamental to describe the epidemiological profile and, consequently, plan preventive actions, in addition to contributing to the development and reformulation of strategies aimed at reducing morbidity and mortality related to suicidal behavior.

## Introduction

As the 13^th^ leading cause of death worldwide, suicide is an important public health problem [1]. In 2016, approximately 817,000 suicides occurred globally, representing 1.49% of total deaths in the world. The estimated mortality rate for all ages was 11.1 deaths per 100,000 individuals. The rate of Potential Years of Life Lost (PYLL) by suicide was estimated to be 458.4 PYLL per 100,000 individuals in the same year, representing 2.18% of the total PYLL [2]. In Brazil, suicide is the fourth cause of death among external causes, and the mortality rate increased from 3.5 (1991) to 5.3 (2015) deaths per 100,000 individuals [3].

Suicide is the act of intentionally causing one’s own death. Although deaths by suicide are rare, suicide attempts and suicidal ideation (thoughts of killing oneself or the desire to die) are more frequent. Self-mutilation is the broader term that includes suicide attempts and self-harm behavior without the conscious intent to cause one’s own death [4]. Suicide attempts consist of “a nonfatal self-directed potentially injurious behavior with any intent to die as a result of the behavior. A suicide attempt may or may not result in injury” [5]. The World Health Organization (WHO) proposes the term “nonfatal suicidal behavior” for suicidal acts that do not end in death since not all suicide attempts end in death. Suicide attempts are an important predictor of death by suicide [6], with the risk being higher for people who have attempted suicide than for those who have not [7, 8]. Approximately 60% of people die by suicide on their first attempt, and more than 80% of subsequent suicides occur within a year of the initial attempt [9].

The prevention of nonfatal suicide attempts is an opportunity for early intervention in a substantial number of people at high risk of suicide to reduce suicidal behavior [10, 11]. In recent years, many studies on suicide-related outcomes (including mortality, attempts, risks, and other measures) have been published worldwide [6]. However, according to the WHO, the quality of data needs to be increased to improve suicide and suicide attempt monitoring and surveillance. There is poor global availability and quality of data on suicide and suicide attempts [12]. Better suicide and suicide attempt monitoring and surveillance are needed for effective suicide prevention strategies. This includes vital statistics records on suicide, hospital records on suicide attempts, and nationally representative surveys that collect information on self-reported suicide attempts [12].

Suicide prevention is a priority for the global public health agenda. It is part of the WHO Mental Health Action Plan 2013–2030. One of the global targets is to reduce the suicide rate by one-third by 2030; to this end, signatory countries should develop and implement comprehensive national prevention strategies, with special attention to groups identified as higher risk and to other vulnerable groups based on the local context [13]. Suicide is also addressed in the Sustainable Development Goals of Target 3.4, which proposes to reduce premature mortality from noncommunicable diseases through prevention and treatment and to promote mental health and well-being by 2030 [14].

Counting cases, monitoring trends by country, and analyzing sex and age distribution are necessary data to support suicide prevention [2]. Accurate and timely data on suicides are necessary for national and international agencies to set targets, assess the progress of interventions, and identify those in need of intervention [12, 15]. However, incorrect cause-of-death classification and quality of data due to the stigmatization of suicide in some countries can affect mortality registries and, therefore, efforts to prevent and reduce mortality from suicide [16].

Although research on suicide has increased in terms of epidemiological profile, risk factors, and temporal trends, few studies analyze the completeness of suicide mortality data, and this number is even lower for suicide attempts. In this study, we analyzed the completeness of data on suicide attempts and suicides included in the surveillance system. Completeness is one of the most important criteria for measuring the quality of datasets in health information systems. It is the degree to which variables are filled with complete information and a category other than the one indicating “missing data” [17].

Strategies aimed at reducing morbidity and mortality associated with suicidal behavior require reliable data [12, 18]. Therefore, assessing the quality of data on suicides and suicide attempts is essential for developing public health and mental health interventions and for planning prevention strategies [18]. Currently, few studies correlate suicide attempts and suicides in Brazil to assess the quality of completeness. Studying the quality and completeness of official records is very important. Well-conducted research on this topic provides key insights for the development of future studies, including the planning of effective evidence-based health policies. Data linkage is a method used to relate variables when the study analyzes more than one database. Complete datasets within a surveillance system are an important prerequisite for local decision-making on planning and prevention strategies. The objective of this study was to analyze the completeness of self-harm and suicide records in Pernambuco, Brazil from 2014 to 2016.

## Methods

### Study design, site, population, and period

This was an evaluative and descriptive study using secondary data regarding the completeness of variables in interpersonal violence, self-harm, and suicide reports of residents of the state of Pernambuco, Brazil. In 2016, the state, which is located in the northeast region of Brazil,had an estimated population of 9,410,336 inhabitants, a territorial area of 98,312 km^2^, and 185 municipalities. The mean suicide mortality rate in the state was 4.7 per 100,000 individuals in 1996–2015, slightly higher than that of the northeast region, which was 4.5 per 100,000 individuals in 2015 [19, 20]. The region was selected for the study for presenting good-quality data in the Mortality Information System (SIM) but needs to improve in suicide data because it reports about 10% of deaths from external causes with undetermined intent [21].

Self-harm is intentional harm to oneself and includes suicidal ideation, self-mutilation, suicide attempt, and suicide [22]. However, suicidal ideation is not recorded in the Notifiable Diseases Information System (SINAN) but requires comprehensive health care actions [22–24]. Suicide attempt is defined as any intentional injury in which there is evidence of suicidal intent but is not fatal [25]. Suicide is the intentional act of killing oneself [19, 26]. All self-inflicted deaths with intent to die were considered suicide according to the International Classification of Diseases—10th Revision (ICD10), codes X60 to X84. The study period (2014–2016) corresponded to the period of the three most recent years with available and completed data in SINAN and SIM.

### Data sources and record linkage

In Brazil, the self-harm notification is mandatory through the use of the individual notification form for interpersonal violence/self-harm, which feeds the SINAN. The tool used to collect data on suicides is the death certificate, which feeds the official SIM. These information systems integrate the strategic efforts of suicide surveillance and prevention in the country.

Database linkage can be defined as the process of merging information from two different sources to determine whether two or more records refer to the same person [27]. In addition, it can be used to find duplicate individual records and in studies using capture and recapture methods [28]. Linkage algorithms must be used when an identifier field is not available [28, 29]. Probabilistic record linkage uses approximate comparison functions. Different weights are assigned to each field based on its distinctiveness and propensity score. This method was used to link data on self-harm (SINAN) and suicide (SIM) since the databases share no common identification field. In this type of linkage, several non-exclusive keys are used to link two pieces of information, such as first and last name [29].

The connections between suicide and self-harm databases were established by a probabilistic relationship using the RecordLinkage package, version 0.4–12.1, within the R software, version 4.0.2. In this study, the variables “name” and “mother’s name” were used for the probabilistic relationship, excluding records in which the nominal identifying fields were ignored or blank.

Scores between zero and one were generated, and linkage suggestions were considered if this score was higher than 0.9. This was defined as a conservative cutoff (which would certify fewer matches) because a manual review was subsequently performed. There is no standard cutoff report for this technique in the literature, and each researcher must make their own choice [30]. After finding match suggestions by linkage, a manual review was performed to exclude false-positive matches.

In the study period (2014 to 2016), 6,594 self-harm notifications (SINAN database) were analyzed, of which 1,404 were suicide attempts. We analyzed 1,219 deaths (SIM database), of which 1,050 were suicides and 169 deaths of undetermined intent.

**Fig. 1:**
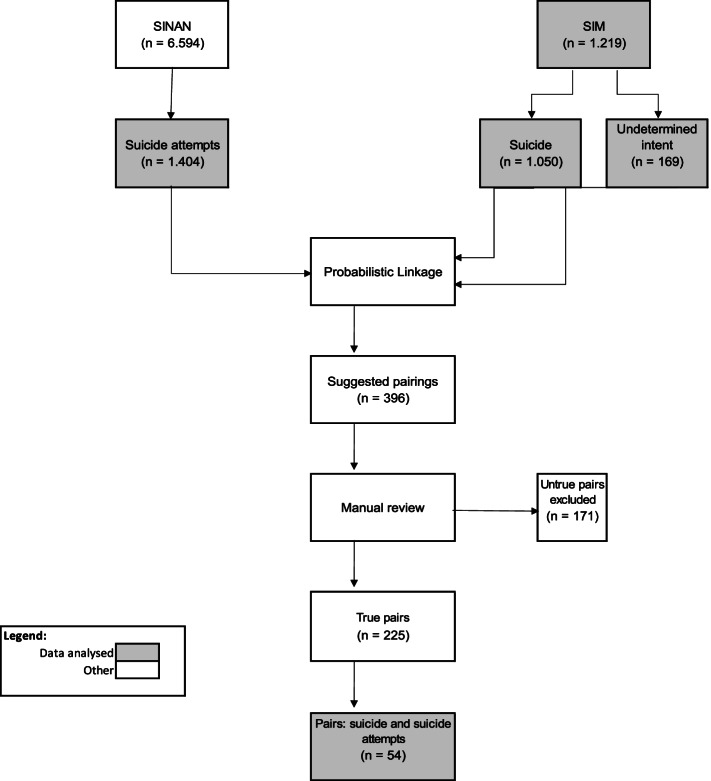
Flowchart of the probabilistic linkage processes performed between the records of the SINAN and SIM

The probabilistic successive relationship identified 396 common records in the two databases (pairs). Of these, 171 were excluded for being false positives, leaving 225 true pairs, 54 of which were suicide reports also identified as suicide attempts. Figure 1 shows the flowchart of the data retrieval process.

### Statistical analysis

The quality of vital statistics data includes several dimensions, including completeness, reliability, and consistency of variables [31]. Data completeness is one of the most analyzed dimensions because the presence of ignored or unfilled variables affects the reliability of these data and, consequently, the knowledge of the real situation of suicides. Completeness was defined as the degree of completion of the fields of the computerized forms analyzed, measured as the proportion of reports in which a field was filled with a different category from the missing data indicators (unknown or blank).

We calculated the degree of completeness of 14 variables from the SINAN to assess the quality of self-harm records: date of notification, date of violence occurrence, date of birth, age, sex, race/color, education, municipality of residence, occupation, marital status, municipality of occurrence, recidivism, means of aggression (physical force/stabbing, hanging, blunt object, sharp object, substance/hot object, poisoning, firearm, threat, other), and place of occurrence. We also analyzed 12 variables from the SIM to assess the quality of suicide records: date of death, age, sex, race/color, marital status, education, occupation, place of residence, municipality of occurrence, place of occurrence, medical care, and autopsy. These variables were selected for their importance in establishing the epidemiological profile of suicide attempts and suicides. In addition, some common variables in both databases allowed the comparison of completeness before and after linkage.

The following classification was used to assess the degree of completeness of the variables: good (≥ 75.1%), regular (50.1–75.0%), low (25.1–50.0%), and very low (≤ 25.0%) [32]. This completeness classification was chosen for being recommended by the Brazilian Ministry of Health in the SINAN Guidelines [24]. For comparison purposes, the same classification was used with the SIM.

The degree of completeness was analyzed before and after linkage for variables common to the suicide attempt and suicide databases. Significant completeness changes between the first and last year of the study were also analyzed. This was also done after linkage, and Pearson’s chi-square test was used to compare proportions. The significance level was set at 5% in this study. Therefore, a *p*-value < 0.05 was considered statistically significant.

### Ethical considerations

The ethical aspects involved in this research were based on the guidelines and standards for human research of Resolution no. 466/2012 of the National Health Council (CNS). The research requires no consent form since it used secondary data; however, the attached risk and confidentiality form was signed. The databases were made available by the Health Secretary of the state of Pernambuco. Only team members had access to the nominal databases, and extreme care was taken to ensure secrecy and confidentiality of information. The data were provided with a letter of consent from the Health Secretary of the state of Pernambuco. The study project was approved by the Research Ethics Committee of the Federal University of Pernambuco (CAEE 17,036,719.0.0000.5208).

## Results

In the period studied, 1,404 notifications of self-harm classified as suicide attempts were analyzed, with an overall mean completeness of 86.2%, and 1,050 suicide records were analyzed, with an overall mean completeness of 95.8%.

Considering the last year of the SINAN database for self-harm by suicide attempt (Table 1), ten variables showed good completeness, two variables (“marital status” and “occurred on other occasions”) had regular completeness, and two other variables (“education” and “occupation”) showed low completeness. The comparison of proportions between the first and last years showed statistically significant improvement in the degree of completeness for four variables (“date of birth,” “race/color,” “municipality of occurrence,” and “suicide attempt: force/courses”). The variables “occupation” and “marital status” showed a decreased proportion. The analysis of the degree of completeness of the variables in the suicide records of the SIM database (Table 1) showed that all variables were classified as good (considering the last year). The comparison of proportions showed that five variables had a statistically significant increase in completeness (“marital status,” “education,” “occupation,” “place of occurrence,” “medical care,” and “autopsy”).

**Table 1:** Completeness of self-inflicted violence variables notified in SINAN and suicides notified in SIM, Pernambuco, Brazil, 2014 to 2016

Variables	2014 (*N* = 387)			2015 (*N* = 427)			2016 (*N* = 590)			*p*-value*	Mean completeness	Variation (%)
	**N**	**%**	**C**	**N**	**%**	**C**	**N**	**%**	**C**			
SINAN												
Notification date	387	100.0	G	427	100.0	G	590	100.0	G	-	100.0	0.0
Date of harm	387	100.0	G	427	100.0	G	590	100.0	G	-	100.0	0.0
Date of birth	360	93.0	G	400	93.7	G	570	96.6	G	0.02	94.4	3.6
Age	387	100.0	G	427	100.0	G	589	99.8	G	1.00	99.9	-0.2
Sex	387	100.0	G	427	100.0	G	590	100.0	G	-	100.0	0.0
Race/color	311	80.4	G	355	83.1	G	506	85.8	G	0.03	83.1	5.4
Education	165	42.6	L	202	47.3	L	261	44.2	L	0.67	44.7	1.6
Municipality of residence	387	100.0	G	427	100.0	G	590	100.0	G	-	100.0	0.0
Occupation	222	57.4	F	225	52.7	F	257	43.6	L	0.00	51.2	-13.8
Marital status	313	80.9	G	352	82.4	G	400	67.8	F	0.00	77.0	-13.1
Municipality of occurrence	334	86.3	G	427	100.0	G	589	99.8	G	0.00	95.4	13.5
Recurrence	244	63.0	F	277	64.9	F	407	69.0	F	0.06	65.6	5.9
Attempts methods (body strength/beating	337	87.1	G	417	97.7	G	565	95.8	G	0.00	93.5	8.7
Place of occurrence	347	89.7	G	372	87.1	G	510	86.4	G	0.16	87.7	-3.2
SIM												
Date of death	382	100.0	G	382	100.0	G	455	100.0	G	-	100.0	0.0
Age	379	99.2	G	381	99.7	G	455	100.0	G	-	99.7	0.8
Sex	382	100.0	G	382	100.0	G	455	100.0	G	-	100.0	0.0
Race/color	379	99.2	G	375	98.2	G	454	99.8	G	0.50	99.0	0.6
Marital status	342	89.5	G	369	96.6	G	446	98.0	G	0.00	94.7	8.5
Education	302	79.1	G	331	86.6	G	418	91.9	G	0.00	85.9	12.8
Occupation	282	73.8	F	314	82.2	G	384	84.4	G	0.00	80.1	10.6
Municipality of residence	382	100.0	G	382	100.0	G	455	100.0	G	-	100.0	0.0
Municipality of occurrence	382	100.0	G	382	100.0	G	455	100.0	G	-	100.0	0.0
Medical care	270	70.7	F	266	69.6	F	388	85.3	G	0.00	75.2	14.6
Necropsy	309	80.9	G	293	76.7	G	414	91.0	G	0.00	82.9	10.1
Place of occurrence	379	99.2	G	382	100.0	G	454	99.8	G	0.50	99.7	0.6

*C* Classification, *G* Good (≥ 75.1%), *F* Fair (50.1 to 75.0%), *L* Low (25.1 to 50.0%), *VL* Very low (≤ 25.0%)

^*^p-value: Pearson’s Chi-squared test for comparison of proportions

Table 2 compares completeness of the variables before and after linkage between the suicide and self-harm databases. The variable “occupation” showed a significantly improved level of completeness.

**Table 2:** Degree of completeness of variables common to SINAN and SIM before and after linkage. Pernambuco, Brazil, 2014 to 2016

Variables	Completeness				*p*-value*	Variation (%)
	**Before**		**After**			
	**N**	**%**	**N**	**%**		
Sex	54	100.0	54	100.0	-	0.0
Age	54	100.0	54	100.0	-	0.0
Race/Color	46	85.2	47	87.0	1.00	1.9
education	21	38.9	23	42.6	0.84	3.7
Occupation	25	46.3	51	94.4	0.00	48.1
Marital status	40	74.1	40	74.1	-	0.0
Municipality of occurrence	54	100.0	54	100.0	-	0.0
Municipality of residence	54	100.0	54	100.0	-	0.0
Place of occurrence	46	85.2	46	85.2	-	0.0

^*^p-value: Pearson’s Chi-squared test for comparison of proportions

## Discussion

The analysis of data on self-harm showed that the completeness of variables was mostly good. It is important to highlight that, since 2016, the immediate notification of self-harm and poisoning is mandatory in Brazil within the first 24 h after the event [22]. This fact compromises the role of SINAN in supporting strategies for monitoring violence [33].

This notification aims to ensure comprehensive care to victims and the link between the health and social protection networks, in addition to strengthening the system for monitoring violence and accidents [22]. The completeness of the data is extremely important to analyzed factors associated with violence and brings subsidies for the design and implementation of actions, programs, and public health policies that improve self-harm prevention [34], similar to the WHO Mental Health Action and Intervention Guide programs [35]. The qualification of notification strategies improves the clinical and psychosocial monitoring and follow-up of cases of attempted suicide in the daily life of services.

The variables related to individual notification (sex and age) were the most complete, as already demonstrated in other studies [33, 36]. These variables are easy to obtain and less subjective, except for the fact that the variable “sex” is mandatory to include a case in the SINAN [22]. However, information on some other variables (“education” and “occupation”) require more attention.

Occupation is directly related to the socioeconomic level of the subject [37] and, due to its association with suicide, this variable becomes relevant and necessary for the study of factors associated with this event [37–39]. People from lower socioeconomic classes and those whose social class has drastically changed have a higher suicide rate than people from higher socioeconomic classes [37, 40].

To have high quality, a database must have complete and reliable records. In the SIM, 100% of the data were complete for the variables “date of death,” “age,” “sex,” “place of residence,” and “place of occurrence.” The completeness was good for the other variables. In addition, an increasing degree of completeness was observed for SIM variables. The complete record of SIM variables increases knowledge on the extent of mortality by suicide so that the information can support intervention planning and evaluation and public policies aimed at suicide surveillance and prevention [12].

In Brazil, the recently created National Policy on Self-harm and Suicide Prevention aims to promote the reporting of events, development and improvement of self-harm, suicide attempt, and death by suicide data collection and analysis methods to help policy creation and decision-making [41].

The overall completeness of the SIM variables was 95.8%. A recent study analyzed these variables and determined an overall completeness of 98.0%, with differences between Brazilian states and municipalities [28]. For deaths from non-natural causes, the general percentage of completeness was 97.8%, but the study did not specifically analyze suicides. In the state of Pernambuco, completeness was greater than 96.0% with most municipalities between 95.0 and 100.0% [25].

In the last years evaluated, the forms were filled more completely, and only the variables “occupation” and “medical care” were below 90% completeness. Previous studies of suicides in the SIM showed progressively increased form filling quality [16, 42, 43]. The completeness of SIM data on suicides of the older population in the period 1996–2010 in Bahia showed improved completeness but still with a large number of ignored or unfilled fields [42]. The evaluation of the quality of information on deaths from external causes in the states of Ceará and Pernambuco also improved [43, 44]. In the latter case, the variable “education” showed good completeness in all the years evaluated. This differs from the situation in this state at the beginning of the 2000s, when the completeness of this variable was considered very poor. The variables “sex” and “place of occurrence” presented good/excellent completeness, confirming our results [44].

The variable “medical care” was also cited in other studies as a cause of lower completeness of death certificates [43, 44]. This field refers to the continuous medical care that the patient received or did not receive during the illness that led to death. The Brazilian Ministry of Health recommends that accidental and/or violent deaths on public roads and without medical care on site should be filled with “no” in this variable. Despite the perception of form filling improvement in this study, 15% remained ignored or were not completed. Further analysis of death certificates, emergency service instruments, and autopsy reports from the Forensic Medicine Institute is necessary to clarify this finding.

The variable “autopsy” showed an improved level of completeness and higher than 90% in the last year of the study. Because there is an Institute of Forensic Medicine in the city of Recife, the question remains whether the variable “autopsy” was not completed or if the deaths by suicide were not referred to the institute. Specialized examinations and autopsies are essential to prevent “hidden suicides” and avoid unclear or unknown causes of death, events of undetermined intent, and accidental deaths [16, 18]. These deaths refer to cases without medical care and those in which there was care, but it was not possible to determine the underlying cause of death or the physician observed only a symptom or sign. They belong to Chapter XVIII of the International Statistical Classification of Diseases and Related Health Problems, 10th revision. In addition, there are deaths with nonspecific causes belonging to several ICD chapters considered of little importance to public health [45, 46].

The need for reliable data on suicides is obvious, but it is one of the most underreported causes of mortality worldwide [18]. Explanations for insufficient data on suicides involve problems related to the reliability of vital statistics records in several countries [47].

A systematic review of the reliability of suicide records found few articles (*n* = 31) and only a few countries were analyzed [48]. This assessment is considered a complex task in cases of suicide. Several issues are raised to explain the difficulties, with some suicides not being properly recorded and some reports being influenced by the subjective interpretations of a person’s death [48]. In addition, the social, cultural, religious, and even legal aspects of a death by suicide may contribute to underreporting [2, 34].

In this study, the probabilistic linkage between self-harm and suicide reports was used as one of the strategies to improve the data. Considering its easy implementation, low operational cost, and reproducibility of the technique, it should be included in the routine monitoring of suicides in the services of the Unified Health System. The linkage of databases from different sources is one of the strategic actions for suicide surveillance and prevention in Brazil [49]. The accuracy and quality of data on suicides are important to identify the circumstances of deaths and describe the victim’s profile. They can affect the planning of preventive actions and the creation of public and mental health policies. From this perspective, continued efforts are needed to improve the accuracy of data, especially in low- and middle-income countries [2, 47].

Improving mortality estimates involves three main issues: the completeness of records, quality of data on causes of death, and production of mortality statistics [50]. To further improve the records of suicide attempts and suicides, some strategies can be suggested, including training health professionals in completing the notification form, monitoring the quality of the database, and raising the awareness of data processing professionals. As for the SIM, it is necessary to train physicians on how to correctly fill out death certificates, monitor data quality, investigate multiple sources, and link information to the suicide attempt database.

This study had some limitations. It is worth noting the use of secondary data, which included typos in the names that were the variables of linkage between the self-harm and suicide databases and classification errors that can lead to linkage process errors. Similar name, sex, and date of birth may also limit linkage. This limitation was minimized by the manual verification of matches and mismatches. The lack of mandatory notification of self-harm in the SINAN may have contributed to the loss of some cases, which was also minimized by the manual review. The study was conducted in a city in northeast Brazil, and cross-cultural limitations should be considered when generalizing the results. However, these limitations did not affect the data analysis and the results of the study.

## Conclusions

Knowledge on the situation of self-harm and suicide mortality trends depends fundamentally on the quality of the data recorded and the monitoring and analysis of the information produced in order to plan actions and define priorities, guidelines, and policies. Therefore, detailing the fields of SIM and SINAN data collection instruments and focusing on their proper registration allow technical knowledge and assure qualified, timely, and reliable information.

## Data Availability

We declare that the datasets used and analyzed during the current study are available at DATASUS.

## Abbreviations

CAEE: Ethics Appreciation Submission Certificate
SINAN: Notifiable Diseases Information System
SIM: Mortality Information System
WHO: World Health Organization
PYLL: Potential Years of Life Lost
